# New Insight into Time-Temperature Correlation for Polymer Relaxations Ranging from Secondary Relaxation to Terminal Flow: Application of a Universal and Developed WLF Equation

**DOI:** 10.3390/polym9110567

**Published:** 2017-11-02

**Authors:** Yonggang Shangguan, Feng Chen, Erwen Jia, Yu Lin, Jun Hu, Qiang Zheng

**Affiliations:** 1 MOE Key Laboratory of Macromolecular Synthesis and Functionalization, Department of Polymer Science and Engineering, Zhejiang University, Hangzhou 310027, China; juhuatai@zju.edu.cn (F.C.); jiaerwen@zju.edu.cn (E.J.); linyu@ecust.edu.cn (Y.L.); 2 The Affiliated Stomatology Hospital, College of Medicine, Zhejiang University, Hangzhou 310006, China; arzthujun@zju.edu.cn

**Keywords:** time-temperature superposition, relaxation, flow, developed WLF equation

## Abstract

The three equations involved in the time-temperature superposition (TTS) of a polymer, i.e., Williams–Landel–Ferry (WLF), Vogel–Fulcher–Tammann–Hesse (VFTH) and the Arrhenius equation, were re-examined, and the mathematical equivalence of the WLF form to the Arrhenius form was revealed. As a result, a developed WLF (DWLF) equation was established to describe the temperature dependence of relaxation property for the polymer ranging from secondary relaxation to terminal flow, and its necessary criteria for universal application were proposed. TTS results of viscoelastic behavior for different polymers including isotactic polypropylene (*i*PP), high density polyethylene (HDPE), low density polyethylene (LDPE) and ethylene-propylene rubber (EPR) were well achieved by the DWLF equation at high temperatures. Through investigating the phase-separation behavior of poly(methyl methacrylate)/poly(styrene-*co*-maleic anhydride) (PMMA/SMA) and *i*PP/EPR blends, it was found that the DWLF equation can describe the phase separation behavior of the amorphous/amorphous blend well, while the nucleation process leads to a smaller shift factor for the crystalline/amorphous blend in the melting temperature region. Either the TTS of polystyrene (PS) and PMMA or the secondary relaxations of PMMA and polyvinyl chloride (PVC) confirmed that the Arrhenius equation can be valid only in the high temperature region and invalid in the vicinity of glass transition due to the strong dependence of apparent activation energy on temperature; while the DWLF equation can be employed in the whole temperature region including secondary relaxation and from glass transition to terminal relaxation. The theoretical explanation for the universal application of the DWLF equation was also revealed through discussing the influences of free volume and chemical structure on the activation energy of polymer relaxations.

## 1. Introduction

As one of the most important issues in polymer science, the time-temperature superposition (TTS) principle is significant and widely used because it describes the time-temperature equivalence for polymer relaxations ranging from secondary relaxations, segmental relaxation to terminal flow, which extends the test range both in experiments and in practice. According to the TTS principle, a master curve concerning the relaxation properties of a polymer at the reference temperature can be obtained by horizontal and vertical shifting of other curves at other temperatures [1]. Moreover, the relationship between shift factors of all curves and temperatures can be described by some mathematical formulas, i.e., the Williams–Landel–Ferry (WLF) equation [2,3] or the Vogel–Fulcher–Tammann–Hesse (VFTH) equation [4,5] for segment dynamics in glass-forming polymer liquids and the Arrhenius equation for secondary relaxations [6] or terminal flow of polymers [7]. In view of its great significance, the TTS principle and the three equations describing the relaxation properties of polymers have attracted many researchers′ attentions [8,9,10,11,12,13,14,15,16,17,18].

By using the WLF equation, Wise et al. [11] calculated the diffusion rate constant during cure and obtained the chemical rate constant for the reaction by the Arrhenius equation in epoxy-amine resins. It was suggested that the WLF constant, *C*_2_, was much less than the universal value under the timescale of their experiments. According to the fact that the temperature dependence of segment diffusion and diffusion-controlled reaction in polymer systems can be described by the WLF equation [8,11], we have put forward an approach to obtain the equilibrium spinodal temperature by introducing a WLF-like equation to analyze the data obtained by small-angle laser light scattering (SALLS) during phase-separation of poly(methyl methacrylate)/poly(styrene-*co*-acrylonitrile) (PMMA/SAN) [13]. However, it has been pointed out that the TTS principle is only valid for miscible polymer blends without a specific interaction when the glass transition temperature (*T_g_*) difference between the constituent components, Δ*T_g_*, is no more than 25 K, whereas it failed for the polymer blends with large Δ*T_g_* [9,10,12]. Furthermore, it was found that the temperature dependence of the phase-separation kinetics of the poly(*n*-methyl methacrylamide)/poly(vinylidene fluoride) (PMMI/PVDF) blend, which is a crystalline/amorphous polymer blend with a large Δ*T_g_* of about 200 K, followed the TTS principle at the early stage and may be described by a WLF-like equation [16]. Liu et al. [14] have found that the VFTH temperature happens to be the melting temperature of quadruplex deoxyribonucleic acid (DNA) by first successfully applying the VFTH equation to biological kinetics to interpret the temperature dependence of the hydrogen exchange rates for the thrombin binding aptamer. In the field of organic liquid electrolytes, the conductivity of a thermally-activated ion-transport process is usually described by the Arrhenius equation in an amorphous phase below *T_g_* [17]. However, above *T_g_*, the temperature dependence of conductivity is described by the WLF equation or the VFTH equation. 

On the other hand, it is well accepted that the three equations mentioned above have their own application limitations. The WLF equation is valid at temperatures ranging from *T_g_* to *T_g_* + 100 K for amorphous polymers [1,2]. The VFTH equation equivalent to the WLF equation is also applicable to describe the relaxations of segments in glass-forming liquids [19,20], while the Arrhenius equation for other relaxations [6,7]. Because of these limitations, these three equations are restricted on some occasions, especially in the overlapping temperature ranges in which relaxations are dominated by different motion units. Plazek [21] had already showed fifty years ago that all the viscoelastic mechanisms contributing to the deformation did not have the same temperature dependence, leading to the difficulty in obtaining a single curve of deformation. However, in his later work [22], it was proposed that the primitive friction factors for the segmental relaxation and terminal relaxation are the same. That means there may be a universal factor that influences segmental and terminal relaxations. By reconciling the transition-state theory [23] and free-volume theory [24], Macedo and Litovitz [25] obtained a good fit for the temperature dependence of viscosity for different liquids over a wide temperature range. Those results show an expectation that exploring a universal equation to describe the relaxations above *T_g_* may be possible based on the same primitive friction factor. Furthermore, considering the relaxation similarity of motion units at different levels for polymers, we try to reveal the internal relationship among the three equations and find a universal form to describe the relaxations over a wide temperature range from both points of experiment and theory, since it is not only significant for polymer theory, but also helpful for the experimental methods and practice.

In this paper, the relationship among the three equations was discussed first from the mathematical point of view. The temperature dependences of viscoelastic behavior for crystallizable polymers and amorphous polymers were examined by rheological measurement. Furthermore, the phase separation behavior of poly(methyl methacrylate)/poly(styrene-*co*-maleic anhydride) (PMMA/SMA) and isotactic polypropylene/ethylene-propylene rubber (*i*PP/EPR) blends detected through SALLS and the secondary relaxations of PMMA and polyvinyl chloride (PVC) measured by broadband dielectric spectroscopy and dynamic mechanical analysis (DMA), respectively, were also investigated. Based on the theoretical analysis, the developed WLF (DWLF) equation was derived from the Arrhenius form. Through discussing the activation energy of polymer relaxations, the theoretical rationality for the DWLF equation was revealed.

## 2. Experimental Section

### 2.1. Materials and Sample Preparation

Commercially available *i*PP (T300, *M*_w_ = 3.83 × 10^5^, *M*_w_/*M*_n_ = 3.34, Shanghai petrochemical, Shanghai, China), EPR (J-0030, *M*_w_ = 1.45 × 10^5^, *M*_w_/*M*_n_ = 2.37; molar percentage of ethylene content is about 45%, Jilin Chemical Industrial Company limited, Jilin, China), high density polyethylene (HDPE) (5000S, *M*_w_ = 1.3 × 10^5^, *M*_w_/*M*_n_ = 5.9, Daqing Petrochemical, Daqing, China), low density polyethylene (LDPE) (LD100BW, *M*_w_ = 9.4 × 10^4^, *M*_w_/*M*_n_ = 5.02, Yanshan Co. Sinopec, Beijing, China), polystyrene (PS) (680A, *M*_w_ = 1.89 × 10^5^, *M*_w_/*M*_n_ = 2.05, Dow, Midland, MI, USA), PMMA (IF850, *M*_w_ = 8.1 × 10^4^, *M*_w_/*M*_n_ = 1.9, LG Co. Ltd., Ulsan, South Korea) and PVC (SG5, Hangzhou Chuanhua Co. Ltd, Hangzhou, China) were adopted. The *i*PP/EPR blend with a composition of 60/40 *wt*/*wt* was dissolved in boiling xylene to form a uniform solution. Then, the blend was obtained by adding the solution into excess methanol to precipitate. The sample was dried in an ambient environment for 48 h and further dried in a vacuum oven at 80 °C for 48 h. Disks of about 1.5 mm in thickness and 25 mm in diameter for rheological measurements were prepared by compression molding under 10 MPa for 8 min at 180 °C for *i*PP, 150 °C for HDPE and 130 °C for LDPE, EPR, PS and PMMA. A small amount of antioxidant was added into the samples during processing.

### 2.2. Small-Angle Laser Light Scattering

A time-resolved small-angle laser light scattering (SALLS) apparatus similar to that described in our previous papers was used [13,26]. The scattering patterns were captured by a Charge Coupled Device (CCD) digital camera (MTV-1802CB, Mintron, New Taipei City, Taiwan), and a He-Ne laser generator (Optical instrument factory of Zhejiang University, Hangzhou, China) was used as the incident beam with the wavelength of 632.8 nm. Some blends between two pieces of cover glasses were placed on a hot stage and melted at 170 °C to form a film of about 140 μm in thickness. Then, the film sample was transported to the hot stage with an appointed temperature from 170 to 237 °C.

### 2.3. Differential Scanning Calorimetry

A differential scanning calorimeter (Q100, TA, New Castle, DE, USA) was used under a protective nitrogen atmosphere. Pure indium and zincum were used as reference materials to calibrate both the temperature scale and the melting enthalpy before the samples were tested. *i*PP samples were first annealed at different temperatures for 30 min and then cooled down to 40 °C with the cooling rate of 10 °C/min. The cooling curves were recorded as the crystallization behavior for *i*PP.

### 2.4. Dynamic Rheological Measurements

The dynamic rheological tests were conducted on an Advanced Rheometric Expansion System (ARES-G2, TA, New Castle, DE, USA) with parallel plate geometry of 25 mm in diameter. The test was performed under conditions as follows: isothermal frequency sweeps from 10 to 0.1 rad/s at different temperatures. A strain of 1%, which was ensured within the linear viscoelastic range, was applied in all tests for *i*PP, HDPE, LDPE and EPR. As for measurements of PS and PMMA, different strains from 0.1% to 1% were adopted.

### 2.5. Dynamic Mechanical Analysis Measurements

Dynamic mechanical analysis (DMA) measurements were carried out on a Q800 analyzer (TA Instruments Corporation, New Castle, DE, USA). Single cantilever mode was used. The measurement was first carried out from −80 °C to 140 °C at a heating rate of 3 °C/min and an oscillatory frequency of 1 Hz. For studying the secondary relaxation, the measurement was carried out from −80 °C to 60 °C with a heating rate of 3 °C/min at different frequencies.

## 3. Results and Discussion

### 3.1. Mathematical Relationship among the Three Equations

The WLF equation was first proposed by Williams et al. to describe the temperature dependence of relaxation mechanisms in amorphous polymers [2]. It is usually expressed in the following form:

(1) $$\mathrm{lg}\alpha_{T}=-\frac{C_{1}\left(T-T_{s}\right)}{C_{2}+\left(T-T_{s}\right)}$$

in which *α_T_* is the shift factor, the ratio of relaxation times at temperatures *T* and *T_s_*, respectively. *T_s_* is the reference temperature, and *C*_1_, *C*_2_ are empirical constants. *C*_1_ = 17.44 and *C*_2_ = 51.6 K are applicable to most amorphous polymers provided *T_g_* is chosen as *T_s_*. A concept, the so-called cooperatively rearranging region introduced by the Adam–Gibbs theory [3] and the free-volume theory [1], has provided the theoretical rationalization for the WLF equation. Another equation equivalent to the WLF function called the VFTH equation is given as [4,5]:

(2) $$\tau =\tau_{0}\exp\frac{B}{T-T_{\infty }}$$

where *τ* is the relaxation time at *T*, *τ*_0_ is a pre-exponential factor, *B* is a numerical constant and *T*_∞_ is the so-called Vogel temperature. Based on the definition of the shift factor, *α*_T_ is the ratio of two relaxation times, which can be expressed as Equation (2), and rearranging the resulting formula algebraically as the WLF form yields [27] (the detailed mathematical derivation is provided in the Supporting Information):

(3) $$C_{1}=\frac{B}{2.303(T_{s}-T_{\infty })}$$

(4) $$C_{2}=T_{s}-T_{\infty }$$

According to the Adam–Gibbs theory, *T*_∞_ is the temperature at which conformational entropy induced by segment motion vanishes and is about 50~60 K below the *T_g_* for most polymers [3]. This fact is consistent with the empirical value of the WLF equation, i.e., *C*_2_ = 51.6 K in the case of *T_s_* = *T_g_*. Moreover, for the WLF and VFTH equations, there are the same temperatures at which the relaxation time is infinity, i.e., *T*_WLF_ = *T*_VFTH_ = *T*_∞_ (*T*_WLF_ = *T_s_* − *C*_2_, *C*_2_ = *T_s_* − *T*_∞_), which accords with the Adam–Gibbs theory.

On the other hand, it is well known that secondary relaxations and terminal flow follow an Arrhenius behavior written as:

(5) $$\tau =\tau_{0}\exp\frac{E_{a}}{RT}$$

in which *E_a_* is the activation energy of relaxation and *R* is the gas constant. Moreover, *E_a_* is the temperature independent activation energy in the Arrhenius form [3]. However, as for real polymers, approaching the glass transition, the apparent activation energy of relaxation will increase significantly [19]. This is the obstacle in essence hindering the application of the Arrhenius equation to glass-forming liquids. Regardless, mathematically, the Arrhenius form is the limit equation of the VFTH form provided *T*_∞_ = 0. Consequently, ignoring the physical meaning of *T*_∞_, the VFTH and WLF form may be universal for glass-forming liquids and terminal flow of polymers. The rationality of *T*_∞_ = 0 for the application of the VFTH or WLF equation at high temperatures will be discussed in more detail later.

Introducing relaxation times of the Arrhenius form into the shift factor, the resulting formula rearranged algebraically as the WLF form can be written as (the detailed mathematical derivation is provided in the Supporting Information):

(6) $$\mathrm{lg}\alpha_{T}=-\frac{0.434\frac{E_{a}}{RT_{s}}\left(T-T_{s}\right)}{T_{s}+\left(T-T_{s}\right)}$$

Introducing two new parameters $C_{1}^{\prime }$ and $C_{2}^{\prime }$,

(7) $$C_{1}^{\prime }=0.434\frac{E_{a}}{RT_{s}}$$

(8) $$C_{2}^{\prime }=T_{s}$$

Then, a developed WLF (DWLF) equation derived from the Arrhenius form can be obtained:

(9) $$\mathrm{lg}\alpha_{T}=-\frac{C_{1}^{\prime }\left(T-T_{s}\right)}{C_{2}^{\prime }+\left(T-T_{s}\right)}$$

Certainly, Equation (9) is applicable to terminal flow of polymers at high temperatures as Equation (5), at least while ${C_{1}}^{\prime }$ = 0.434*E_a_*/*RT_s_*, ${C_{2}}^{\prime }$ = *T_s_*. However, the doubt is whether there are rational values for ${C_{1}}^{\prime }$ and ${C_{2}}^{\prime }$ that are suitable for glass-forming liquids and terminal flow simultaneously. For this purpose, the apparent activation energy of Equation (9) should meet two requirements: (i) it decreases strongly with increasing temperature nearby the glass transition; (ii) it tends to be a temperature independent value at high temperatures far away from the glass transition. For the WLF equation, apparent activation energy can be calculated formally as [1,27]:

(10) $$\Delta E_{a}^{WLF}=R\frac{d\ln\alpha_{T}}{d(1/T)}=2.303RC_{1}C_{2}\frac{T^{2}}{{\left(C_{2}+T-T_{s}\right)}^{2}}$$

Analogously, the apparent activation energy of Equation (9) can be written as:

(11) $$\Delta {E^{\prime }}_{a}=2.303RC_{1}^{\prime }C_{2}^{\prime }\frac{T^{2}}{{\left(C_{2}^{\prime }+T-T_{s}\right)}^{2}}$$

In order to meet the two requirements above, we have:

(12) $$\frac{\partial \left(\Delta E_{a}^{\prime }\right)}{\partial T}\leq 0$$

(13) $$\frac{\partial \left(\partial \left(\Delta E_{a}^{\prime }\right)/\partial T\right)}{\partial T}\geq 0$$

From the calculation of the two inequalities above, the criterion for the universality of Equation (9) can be obtained:

(14) $$C_{2}^{\prime }\leq T_{s}\leq T+C_{2}^{\prime }$$

Consequently, Equation (9) may be universal while the criterion above (Inequality (14)) is met. However, it should be pointed out that when ${C_{2}}^{\prime }$ = *T_s_*, Equation (9) will evolve into the Arrhenius equation according to Equation (6), and the criterion (Inequality (14)) is a necessary, but not a sufficient condition for the universality of Equation (9).

### 3.2. Application of the DWLF Equation for Viscoelastic Behavior of Pure Polymers at High Temperatures

**Figure 1:**
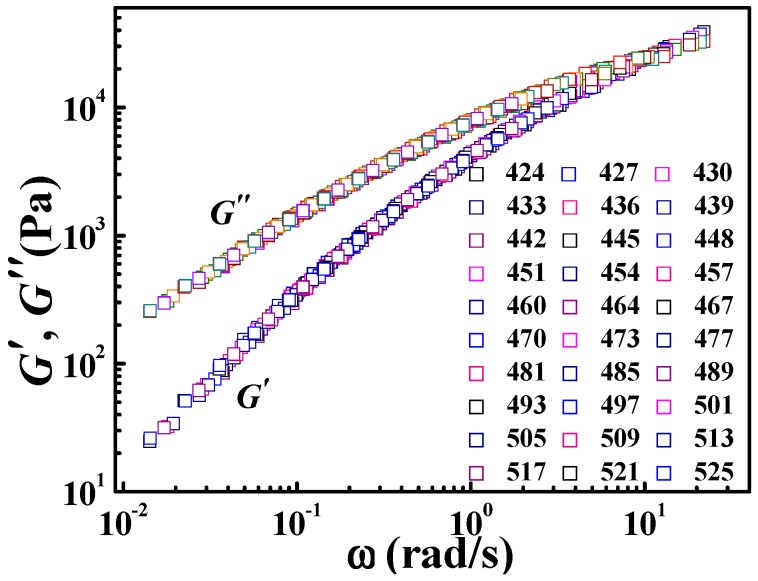
Master curve by horizontal shifting of the frequency (*ω*) dependence of *G*′ and *G*″ at different temperatures for isotactic polypropylene (*i*PP); in which the reference temperature is 454 K.

As mentioned above, the DWLF equation may be applied at high temperatures where the Arrhenius equation is traditionally employed. Here, the viscoelastic properties of various pure polymers (crystalline and amorphous polymer) and polymer blends (amorphous/amorphous blend and crystalline/amorphous blend) were investigated. Figure 1 gives the master curve by horizontal shifting of the frequency (*ω*) dependence of the dynamic storage modulus (*G*′) and dynamic loss modulus (*G*″) for pure *i*PP at temperatures from 424 to 525 K. It is found that all curves obtained at different temperatures can superpose together and then form a satisfactory master curve by horizontal shifting. This result indicates that the TTS principle can be applicable for *i*PP at high temperatures. 

**Figure 2:**
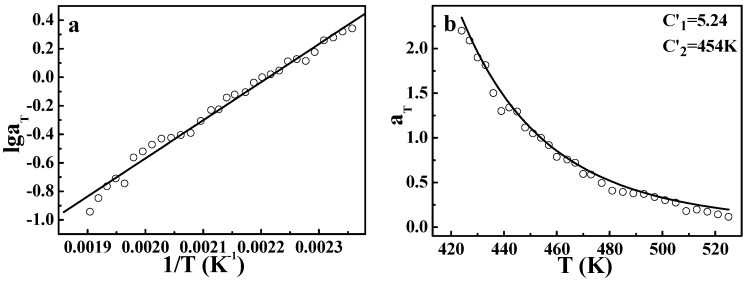
Time-temperature superposition obtained by (**a**) the Arrhenius equation and (**b**) the developed Williams–Landel–Ferry (DWLF) equation for *i*PP. The reference temperature is 454 K.

However, it should be pointed out that from the theory of free volume, the WLF equation is only applicable for amorphous polymers at the temperature range of *T_g_* ~ *T_g_* + 100 K [1,2], since the crystalline phase will certainly restrict the expansion of free volume and change the linear dependence of free volume on temperature. While in the molten state, the limitation of the crystalline phase vanishes, the equation of the WLF form may be applicable again. Based on this understanding and the discussion above, both the Arrhenius equation and the DWLF equation are used to describe TTS of *i*PP, as shown in Figure 2. It is to be expected that both = equations can describe TTS of *i*PP well at high temperatures. Here, *T_s_* = 454 K is chosen as the reference temperature, and the two parameters of the DWLF form are *C*_1_′ = 5.24 and *C*_2_′ = 454 K, respectively. The extreme equivalence of *C*_2_′ to *T_s_* indicates that this result is logical according to the criterion above (Inequality (14)). However, the values of the two parameters deviate from the empirical values of the WLF equation, suggesting that the apparent activation energies of the viscoelastic behavior for different polymers at high temperatures vary widely and may be correlated with a specific chemical structure [28]. This viewpoint is also supported by Williams’ results [2] that the curves log*α_T_* ~ (*T* − *T_s_*) no longer overlap for different polymers at high temperatures. In that case, *C*_1_′ and *C*_2_′ will deviate from the empirical values. These results show that the viscoelastic behavior of *i*PP, a typical crystalline polymer, follows the TTS principle and could be described by the DWLF equation in the molten state, in spite of the deviation from empirical values for *C*_1_′ and *C*_2_′. 

It should be reasonable that the choice of reference temperature is quite arbitrary according to the knowledge of the traditional WLF equation. Table 1 gives the two parameters at different reference temperatures. Additionally, the goodness of fit (GOF) calculated from:

(15) $$R^{2}=1-\frac{\sum {\left(y_{i}-y_{i}^{\prime }\right)}^{2}}{\sum y_{i}^{2}}$$

was used to evaluate the consistency between measured value *y_i_* and calculated value *y_i_*′ from the DWLF equation.

It is clearly seen that for all reference temperatures investigated, the value of *C*_2_′ is equal to that of *T_s_*, meaning that the choice of reference temperature can be indeed quite arbitrary. Moreover, the results of the viscoelastic behavior described by the DWLF equation at other reference temperatures are also perfect (seen in the Supporting Information, Figure S1). Considering that the glass transition temperature of *i*PP is about 288 K (seen in the Supporting Information, Figure S2), the temperature here is much higher than *T_g_* + 100 K, indicating that the application of the DWLF equation at high temperatures is feasible. Furthermore, the apparent activation energy of viscoelastic behavior can be calculated from the Arrhenius equation, i.e., ∆*E_a_* = 51.2 kJ/mol, while that obtained by using the data in Table 1 with the DWLF equation according to Equation (7) is ♦*E_a_*_-WLF_ = 48.1 ± 3.1 kJ/mol. These two values of apparent activation energies are approximate, confirming the effectiveness of the DWLF equation at high temperatures again.

**Figure 3:**
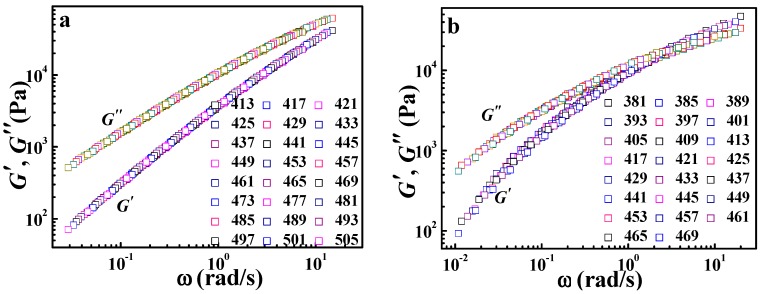
Master curves by horizontal shifting of the frequency (*ω*) dependence of *G*′ and *G*″ for (**a**) high density polyethylene (HDPE) and (**b**) low density polyethylene (LDPE). The reference temperature of HDPE is 433 K, and that of LDPE is 401 K.

**Figure 4:**
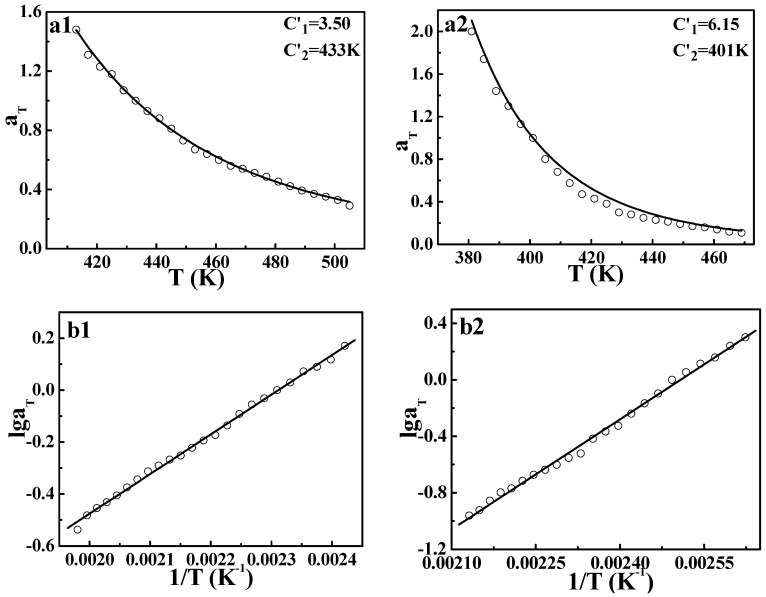
Time-temperature superpositions described by (**a**) the DWLF equation and (**b**) the Arrhenius equation for (1) HDPE and (2) LDPE; in which the reference temperature of HDPE is 433 K and that of LDPE is 401 K.

However, the application of the TTS principle to viscoelastic behavior for crystalline polymers is still rarely reported. In order to further explore the validity of the DWLF equation, another two crystalline polymers, i.e., HDPE and LDPE, were also investigated. The master curves of HDPE and LDPE are displayed in Figure 3. Similar to the case of *i*PP, both HDPE and LDPE show a good overlapping curve. Moreover, the temperature dependences of shift factors described by the DWLF equation and the Arrhenius equation for each polymer are shown in Figure 4. It is found that as a whole, the DWLF equation can describe the relaxation behavior well for them in the molten state similar to the Arrhenius form. It is noted that the fitting curve of the DWLF equation deviates from the experimental data for LDPE at a temperature ranging from 400 to 440 K, as shown in Figure 4(a2). It may be ascribed to the existence of the abundantly branched structure in LDPE, which may lead to some additional relaxation. In addition, the values of parameter *C*_2_′ are equal to the reference temperatures, respectively, according to the criterion above (Inequality (14)). At other reference temperatures, the results are also perfect. Considering the fact that both the glass transition temperatures of HDPE and LDPE are far lower than the temperature range investigated here (166 K and 158 K for *T_g_* of HDPE and LDPE respectively, seen in the Supporting Information, Figure S3), it is reasonable to draw the conclusion that in the molten state, the limitation of the expansion of free volume by the crystalline phase disappears, leading to the application of the DWLF equation. From the calculation, the apparent activation energies of the viscoelastic behavior obtained by the DWLF equation and the Arrhenius form are ♦*E_a_* = 29.0 kJ/mol (the DWLF equation), ♦*E_a_* = 29.3 kJ/mol (the Arrhenius form) for HDPE, ♦*E_a_* = 50.0 kJ/mol (the DWLF equation) and ♦*E_a_* = 49.8 kJ/mol (the Arrhenius form) for LDPE, respectively. Almost the same apparent activation energy obtained by the two equations supports the universality of the DWLF equation for crystalline polymers in the molten state well.

**Figure 5:**
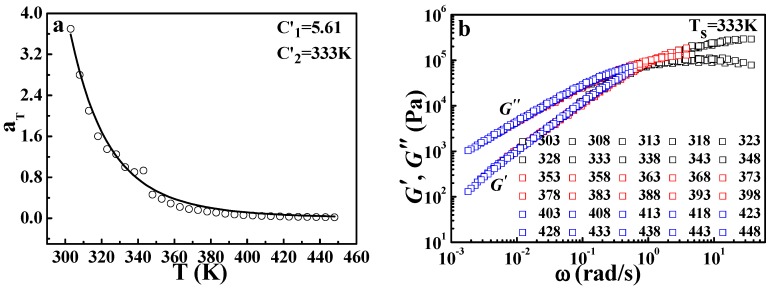
(**a**) Time-temperature superposition described by the DWLF equation for ethylene-propylene rubber (EPR) and (**b**) its master curve; in which the reference temperature is 333 K.

The results of crystalline polymers above have proven that the DWLF equation at a high temperature can be equal to the Arrhenius form provided *C*_2_′ = *T_s_* and can be used to describe the relaxation behavior of crystalline polymers. Consequently, it seems no obstruction for application on the amorphous polymer. Figure 5 shows the TTS results of the viscoelastic behavior of EPR, a rubber with *T_g_* of 233 K (seen in the Supporting Information). Similar to the results of crystalline polymers, the DWLF equation can describe the relaxation behavior of EPR at high temperatures far away from the glass transition range.

### 3.3. Application of the DWLF Equation for the Phase-Separation Behavior of Polymer Blends at High Temperatures

The discussions above have shown that the DWLF equation is applicable to describe the relaxation behavior of pure crystalline and amorphous polymers at high temperatures far above *T_g_*. Our previous work has also proven that the WLF-like equation can perfectly describe the phase-separation behavior of the PMMA/SAN blend investigated by SALLS within the temperature range of *T_g_* ~ *T_g_* + 100 K [13]. Considering the results above, it should be logical that the DWLF equation can also describe the phase separation behavior of polymer blends at higher temperature above *T_g_* + 100 K. Here, the data of the phase separation of the amorphous/amorphous polymer blend (PMMA/SMA) with a lower critical solution temperature (LCST) character, which have been described by the Arrhenius equation in our previous paper [29], were re-examined. The shift factors investigated by SALLS are calculated from:

(16) $$\alpha_{T}=\frac{\tau }{\tau_{s}}$$

**Figure 6:**
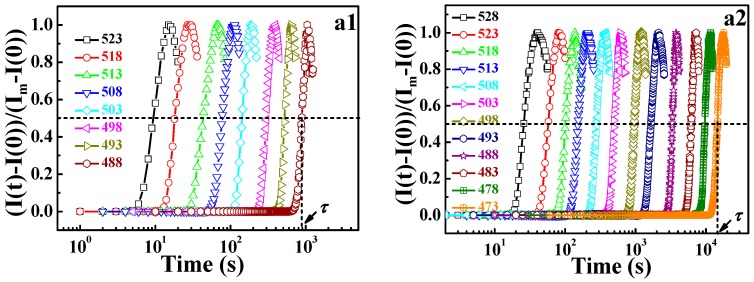

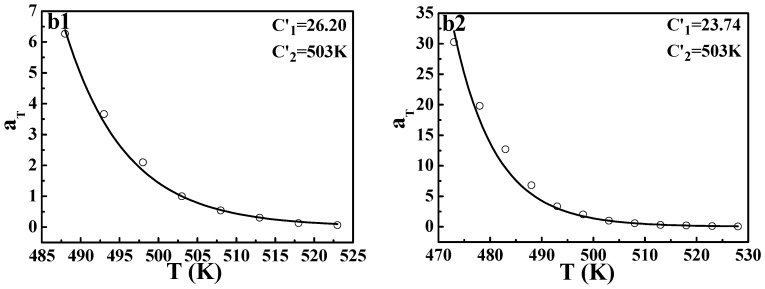
(**a**) Time evolution of normalized scattering intensity and (**b**) DWLF plots of shift factors for two poly(methyl methacrylate)/poly(styrene-*co*-maleic anhydride) (PMMA/SMA) blends: (1) 60/40 at *q* = 5.5 μm^−1^ and (2) 80/20 at *q* = 4.0 μm^−1^ for various temperatures. The data were from our other paper [24]. The reference temperature is 503 K. *T_g_* of PMMA/SMA 60/40 and 80/20 blends were 385 and 378.4 K, as measured using dielectric spectroscopy, respectively [18].

Here, *τ_s_* is the relaxation time at *T_s_*, and *τ* is the relaxation time at which the normalized scattering intensity for different temperatures increases by the same degree, being 50% in this work, as shown in Figure 6a. From Figure 6b, it is seen that the DWLF equation describes the phase separation behavior of different PMMA/SMA blends well. For the two PMMA/SMA blends with different compositions, the apparent activation energies of phase separation obtained by the DWLF equation are ♦*E_a_* = 252.4 ± 3.0 kJ/mol and ♦*E_a_* = 228.8 ± 2.5 kJ/mol, respectively, which are similar to those obtained by the Arrhenius form (♦*E_a_* = 278.2 ± 8.4 kJ/mol and ♦*E_a_* = 241.5 ± 4.9 kJ/mol, respectively) [29]. Considering the fact that liquid-liquid phase separation is a process synchronously containing disentanglement and segment motion, which depend on relaxation time and the glass transition temperatures of PMMA/SMA 60/40 and 80/20 blends, which are 385 and 378.4 K respectively [18], the above results indicate that the DWLF equation can describe the relaxation process, i.e., rheological behavior and phase separation behavior, well at temperatures above *T_g_* + 100 K.

**Figure 7:**
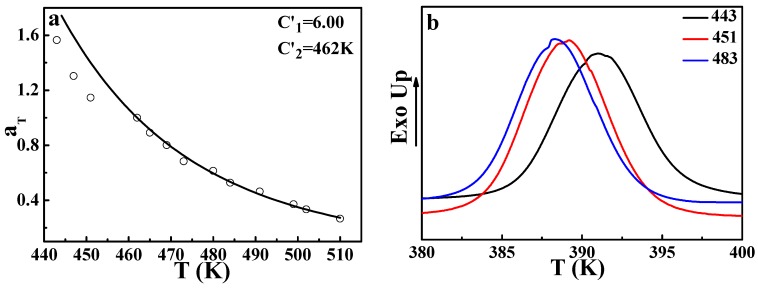
(**a**) DWLF curve of the shift factors of the phase separation behavior investigated by small-angle laser light scattering (SALLS) for the *i*PP/EPR (60/40) blend; in which *T_s_* = 462 K. (**b**) Crystallization behavior of *i*PP after annealing at different temperatures.

In order to further explore the validity of applying the DWLF equation to the phase separation behavior of polymer blends, a crystalline/amorphous polymer blend (*i*PP/EPR) was used for investigating, as shown in Figure 7a. It is found that at low temperatures, the experimental values of *α_T_* deviate from the theoretical curve. However, the experimental values at high temperatures are very consistent with the DWLF curve. Considering that the equilibrium melting point of *i*PP is 460 K [30], *i*PP will crystallize when the temperature is below 460 K in theory. Furthermore, it was pointed out that nucleation behavior may exist, and the lamellar structures of *i*PP can survive in the melt for a long time even if the annealing temperature is above the apparent melting point, *T_m_* [31]. Regardless, the final point of the melting temperature range is 448 K for *i*PP (seen in the Supporting Information). This fact means that at these temperatures, the nucleation of *i*PP may happen, though the crystal growth is extremely slow at temperatures near the equilibrium melting point.

Figure 7b gives the crystallization behavior of *i*PP after being annealed at different temperatures for 30 min. The increase of the crystallization temperature after being annealed at 443 K and 451 K indicates that the nucleation process indeed exists even at a temperature higher than its *T_m_* (439 K, seen in the Supporting Information). It is well known that during the crystallization process, the heterogeneous components can be excluded from the crystalline phase [32]. There is no doubt that this nucleation behavior can lead to a heterogeneous concentration fluctuation. In other words, at these temperatures, heterogeneous concentration fluctuation resulting from nucleation of *i*PP could affect the phase-separation behavior. That means the existence of the nucleation of *i*PP provides an additional contribution to the phase-separation, indicating that the time required for a 50% increase of the scattered light intensity becomes shorter, leading to a smaller *α*_T_ than the theoretical value. This may be the cause of the slight deviation in the melting region.

### 3.4. Universality of the DWLF Equation in a Wide Temperature Range

**Figure 8:**
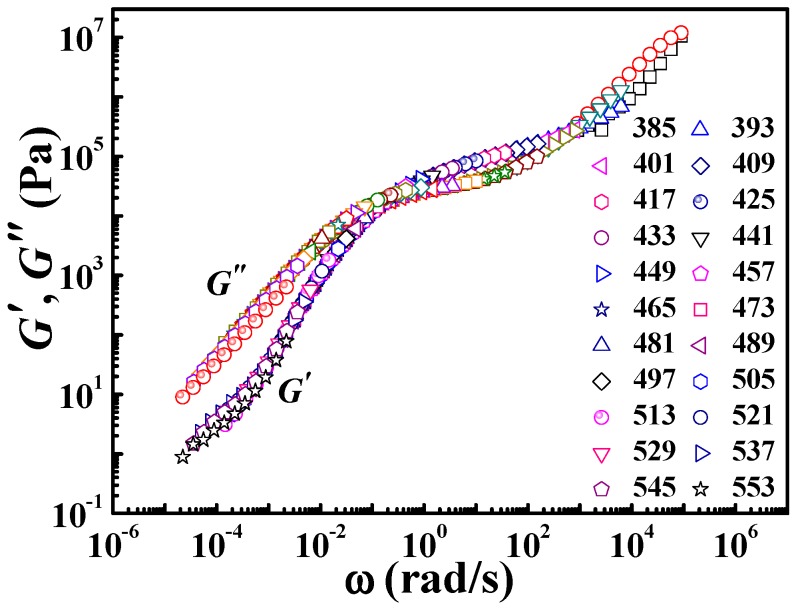
Master curve by horizontal shifting of the frequency (*ω*) dependence of *G*′ and *G*″ at different temperatures for polystyrene (PS). The reference temperature is 425 K.

The above results indicate that the application of the DWLF equation at high temperatures where the Arrhenius equation is traditionally employed is appropriate indeed. Consequently, the universality of Equation (9) at temperatures from glass transition to very high temperatures seems reasonable. Here, due to the suitable *T_g_* (378 K for PS, seen in the Supporting Information), PS was used to examine the universality of Equation (9) from *T_g_* to much high temperatures. *T_s_* = 425 K was chosen as the reference temperature, about 50 K higher than its *T_g_* (378 K). The master curve at the reference temperature can be obtained by horizontal shifting of frequency (*ω*) dependence (from 0.1 rad/s to 10 rad/s) of *G*′ and *G*″ at different temperatures from 385 K to 553 K, as shown in Figure 8. The temperature range here covers both regions, i.e., the vicinity of glass transition and high temperatures far away from *T_g_* + 100 K.

**Figure 9:**
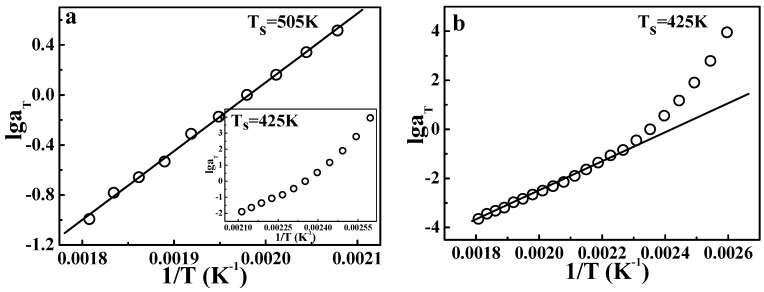
Time-temperature superposition of the shifting factor for PS described by the Arrhenius equation. (**a**) Application to the fractional temperature region; and (**b**) application to the whole temperature region.

Figure 9 gives the results of TTS described by the Arrhenius equation. Figure 9a shows the application of the Arrhenius form at temperatures from 481 to 553 K, and the insert gives the result from 385 to 473 K. Legitimately, the points at high temperatures (481 to 553 K) show a good linear relationship, according to the Arrhenius equation. However, the insert in Figure 9a shows a curve, not a line, indicating that in the vicinity of glass transition, the Arrhenius equation is no longer valid. These results are consistent with our common knowledge. Of course, the Arrhenius equation is invalid in the whole temperature range also, as shown in Figure 9b. This fact also means that at temperatures from 385 to 473 K, the apparent activation energy shows a strong dependence on the temperature.

**Figure 10:**
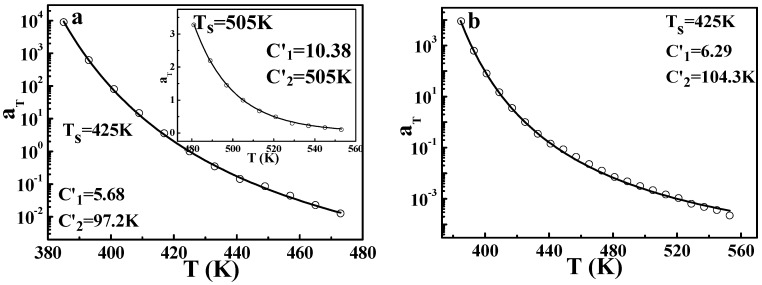
Time-temperature superposition of the shifting factor for PS described by the DWLF equation. (**a**) Application to the fractional temperature region; and (**b**) application to the whole temperature region.

Based on the results and discussions above, the DWLF equation can be applicable at very high temperatures. In addition, the vicinity of glass transition is also the traditional region in which the WLF form is valid. Consequently, it is interesting whether the DWLF equation can be availably applied to the whole temperature range. Differing from that of the Arrhenius form, it is found that the DWLF equation can be applied in both regions well, as shown in Figure 10a. In the vicinity of glass transition (from 385 to 473 K), the two parameters are *C*_1_′ = 5.68 and *C*_2_′ = 97.2 K, differing from the empirical values (*C*_1_ = 8.86 and *C*_2_ = 101.6 K) when the reference temperature is located roughly 50 K above *T_g_* [2]. Additionally, at high temperatures, the DWLF equation also accords with the data perfectly, shown as the insert in Figure 10a, provided *T_s_* = *C*_2_′ according to the criterion above. Moreover, in the whole temperature range, the TTS of the viscoelastic behavior for PS can be described by the DWLF equation excellently again, indicating that Equation (9) is indeed universal for a wide temperature range. In view of the larger value of *C*_2_′ in Figure 10b (*C*_2_′ = 104.3 K) than that in Figure 10a (*C*_2_′ = 97.2 K), this means that due to the higher temperature, the DWLF equation approaches the Arrhenius form more and more. When the temperature region is wholly higher than *T_g_* + 100 K, the value of *C*_2_′ is equal to *T_s_*, shown as the insert in Figure 10a.

**Figure 11:**
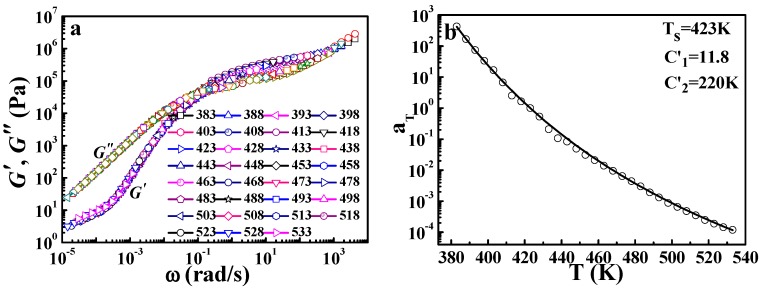
(**a**) Master curve and (**b**) time-temperature superposition described by the DWLF equation of PMMA. The reference temperature is 423 K.

In addition, the TTS of other pure polymers, for example, PMMA, is also investigated. Figure 11a shows the master curve of PMMA at *T_s_* = 423 K (for PMMA, *T_g_* = 370 K, as seen in the Supporting Information). Similar to PS, the master curve of PMMA at the reference temperature can be obtained by horizontal shifting of the frequency (*ω*) dependence of *G*′ and *G*″ at different temperatures. The application of the DWLF equation is displayed in Figure 11b. All the data points show the consistency with the DWLF curve, meaning the DWLF equation is also valid within the wide temperature range from *T_g_* to very high temperatures in this case.

The results above have indicated that the DWLF equation is universal at temperatures above *T_g_*. When the temperature is much higher than *T_g_* + 100 K, the DWLF equation evolves into the Arrhenius form. Based on this concept the Arrhenius form may be expressed as:

(17) $$\tau =\tau_{0}\exp\frac{2.303RC_{1}^{\prime }C_{2}^{\prime }\frac{T^{2}}{{\left(C_{2}^{\prime }+T-T_{s}\right)}^{2}}}{RT}$$

In the vicinity of glass transition, the item *T*^2^/(*C*_2_′ + *T* − *T*_s_)^2^ depends on temperature strongly, meaning a strong temperature dependence of apparent activation energy. When the temperature is far away from the glass transition, *C*_2_′ approaching *T_s_* will result in being one for this item, meaning that the apparent activation energy becomes approximately temperature-independent.

**Figure 12:**
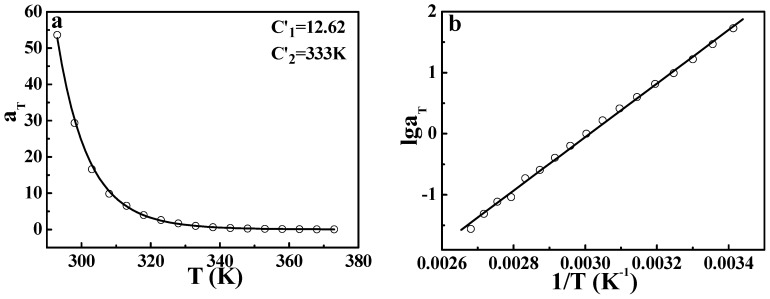
(**a**) DWLF curve and (**b**) Arrhenius line of the *β*-relaxation process for PMMA. The data were from our other paper [18]. The reference temperature is 333 K.

As pointed out in the Introduction, the Arrhenius form can describe both the terminal relaxation and secondary relaxation of polymers. In the discussion above, it has been proven that the DWLF equation can be universal at temperatures above *T_g_*. However, a question arises: is it appropriate to use the DWLF equation on the secondary relaxation of polymers? Based on the equivalence of the DWLF equation to the Arrhenius form at *C*_2_′ = *T_s_*, it seems no obstruction for the application on secondary relaxation. Figure 12 gives the results of the secondary relaxation of PMMA, which has been described by the Arrhenius form in our other paper [18]. It is seen that the DWLF equation can also describe the secondary relaxation well. The apparent activation energy of secondary relaxation obtained by the DWLF equation is ♦*E_a_* = 80.5 ± 0.2 kJ/mol, which is near that obtained by the Arrhenius form (♦*E_a_* = 84.1 ± 0.2 kJ/mol) [18]. Thus, Figure 11 and Figure 12 indicate the fact that the DWLF equation can describe the relaxation behavior of the polymer from secondary relaxations to the terminal flow according to two different reference temperatures: one is for secondary relaxation and the other for those above *T_g_*.

**Figure 13:**
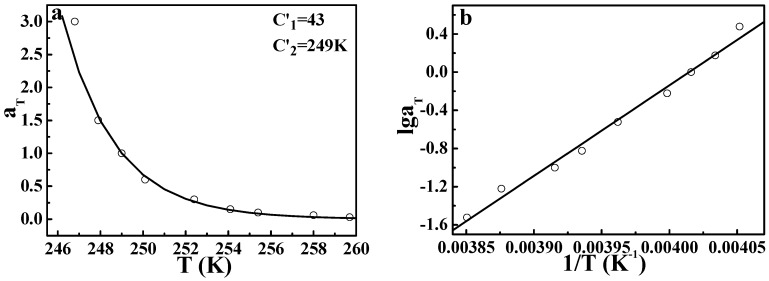
(**a**) DWLF curve and (**b**) the Arrhenius line of the *β*-relaxation process for PVC. The reference temperature is 249 K.

To examine the effectiveness of the DWLF equation in describing secondary relaxations further, the *β*-relaxation process of PVC was also studied. Figure S8 (seen in the Supporting Information) shows that the *β*-relaxation of PVC occurs at about 253 K at 1 Hz. When the frequency decreases, the temperature at which *β*-relaxation occurs also decreases. Therefore, *α*_T_ obtained from different frequencies can be described by the DWLF equation and the Arrhenius form. The results are given in Figure 13. Both equations can describe the *β*-relaxation behavior well. Moreover, the apparent activation energies obtained from the two equations are also approximate, i.e., ♦*E_a_* = 205 ± 6.9 kJ/mol for the DWLF equation and ♦*E_a_* = 181.8 ± 7.2 kJ/mol for the Arrhenius form, respectively.

### 3.5. Theoretical Analysis of the Universal Application of the DWLF Equation

The results and discussions above have shown that the Arrhenius form is the limiting form of the WLF (VFTH) equation provided *T*_∞_ = 0, and the DWLF equation can be applicable in a wide temperature range if there are rational values of *C*_1_′ and *C*_2_′. However, the rationality of *T*_∞_ = 0 is based on ignoring the physical meaning of *T*_∞_. In this section, the operation of *T*_∞_ = 0 will be discussed theoretically.

**Figure 14:**
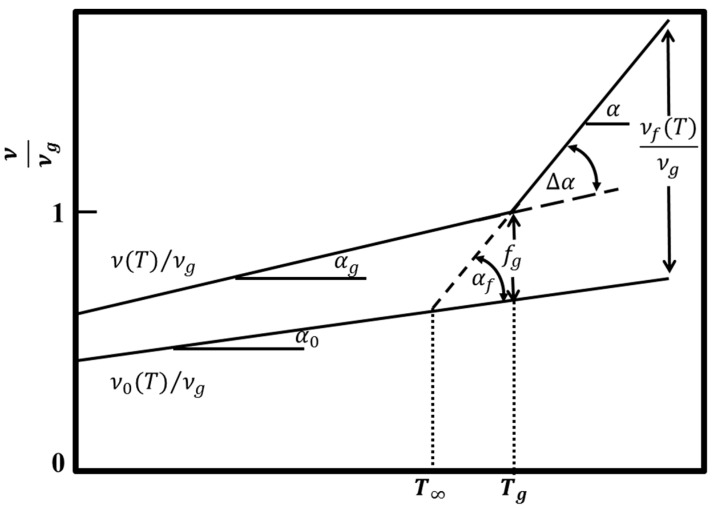
Schematic diagram depicting total specific volume *ν*(*T*), occupied volume *ν*_0_ and free volume *ν_f_* (relative to specific volume at *T_g_*) with temperature for a supercooled liquid [27]; in which *ν_g_* is specific volume at *T_g_*, *α_g_* and *α* represent volume expansion coefficients in the glassy and rubbery states and *α*_0_ and *α_f_* are the expansion coefficients of occupied volume and free volume, respectively. *f_g_* is the fractional free volume (*ν_f_/ν_g_*). ∆*α* is the difference between *α* and *α_g_*.

According to the thermodynamic theory of glass transition by Gibbs and DiMarzio [33,34,35,36], glass transition is indeed a true second-order transition, and the true equilibrium *T_g_* (defined as *T*_2_) can be obtained only at infinitely long times, which is difficult to realize. In infinitely slow experiments, no discontinuity will be observed in the first-order properties (volume and internal energy) at *T*_2_, but the second-order properties (thermal expansion coefficient and heat capacity) will exhibit discontinuous change [37]. The equilibrium *T_g_* (*T*_2_) at which the conformational entropy of the polymer is zero always lies about 50 K below the *T_g_* observed at ordinary times for most polymers [7,38]. On the other hand, Fox and Flory first used the free volume theory to explain the glass transition of polymers [37,39]. The core idea of free volume theory can be described by Figure 14 in which *ν*(T) is total specific volume, *ν*_0_, *ν_f_* is occupied volume and free volume, respectively, and *ν_g_* is specific volume at *T_g_* [1,27]. In this theory, the glass state is an iso-free-volume state for all polymers. In the glass state, thermal expansion is only contributed to by the expansion of occupied volume; while in the rubber state, thermal expansion is contributed to by both occupied volume and free volume. Since segment motion depends on the free volume, at temperatures below *T_g_*, chain segments are frozen and cannot move due to the freezing of the fractional free volume. According to free volume theory, *T_g_* can be determined as the temperature at which the thermal expansion coefficient undergoes a discontinuity during cooling. Considering the fact that the conformational entropy of the polymer is zero at equilibrium *T_g_*, according to the Gibbs–DiMarzio theory [7,33,34,35,36,38], the equilibrium *T_g_* must be the temperature at which the fractional free volume is zero since the conformational entropy can be zero only in the case that there is not any free volume according to the free volume theory [1,37,39]. Moreover, Sperling [7] pointed out that free volume shrinks very slowly with decreasing temperature even in the glass state. If temperature decreases with an infinitely slow rate, the equilibrium fractional free volume can be obtained during the whole experiment, shown as the short dashes in Figure 14 [27]. This equilibrium line of *ν*(*T*)/*ν_g_* will intersect with the line of *ν*_0_(T)/*ν_g_*. It is easy to understand that the fractional free volume at the crossover point is zero from Figure 14. Consequently, the conformational entropy of the polymer at this state is zero since there is not any free volume to realize the movement of chain segments. That means that *T*_∞_ in Figure 14 is the equilibrium *T_g_*. From Figure 14, it is easy to obtain:

(18) $$\left(\alpha -\alpha_{0}\right)\times \left(T_{g}-T_{\infty }\right)=\alpha_{f}\times \left(T_{g}-T_{\infty }\right)=f_{g}$$

Considering the fact that fractional free volume in the glass state is 0.025 for most polymers and *α_f_* = 4.8 × 10^−4^ K^−1^ [1,2], we have [27]:

(19) $$\left(T_{g}-T_{\infty }\right)=\frac{f_{g}}{\alpha_{f}}=52.1\;K$$

That value is consistent with the Gibbs–DiMarzio theory in which equilibrium *T_g_* always lies about 50 K below the apparent *T_g_* [7,38]. In view of the discussions on the VFTH equation in Section 3.1, it is easy to understand that the so-called Vogel temperature *T*_∞_ in the VFTH equation is also just the equilibrium *T_g_* in Figure 14 according to the Adam–Gibbs theory [3]. When *T_g_* is chosen as *T_s_* in the WLF form, it can be concluded that there are universal values of *C*_1_ and *C*_2_ for most polymers according to Equations (3), (4) and (19).

Since the mobility of chain segments depends on the free volume, the temperature dependence of the relaxation for polymers indeed depends on free volume [1]. The free volume dependence of relaxation is successfully applied by Doolittle in his viscosity equation [24,40,41,42,43]:

(20) $$\ln\eta =B\left(\frac{\nu_{0}}{\nu_{f}}\right)+\ln A$$

where *A* and *B* are empirical constants. On the other hand, the diffusion coefficient (*D*) of entangled linear polymers in the molten state can be obtained according to de Gennes [44,45],

(21) $$D=\frac{kT}{\xi }\times \frac{N_{e}}{N^{2}}$$

in which *k* is the Boltzmann constant, *T* is absolute temperature, ξ is the friction coefficient, *N_e_* is the number of Kuhn monomers in an entanglement strand and *N* is the number of Kuhn monomers in a macromolecular chain. Equation (21) indicates that the relaxation of polymers is also influenced by the specific chemical structure. Consequently, it is reasonable to say that the potential energy barrier (*E_a_*) for relaxation should be composed of two items, i.e.,

(22) $$E_{a}=E_{f}+E_{s}$$

in which *E_f_* stands for the contribution of free volume and *E_s_* stands for the contribution of the specific chemical structure. According to the pioneering work of Turnbull and Cohen on free volume theory [46,47], it is pointed out that at small *ν_f_*, considerable energy is required to redistribute the free volume; while when *ν_f_* is larger than a critical value, the free volume can be redistributed freely. When the temperature is near the glass transition, the molecular relaxation rate is strongly limited by the large energy barrier, which is required for redistributing the free volume [47,48]. Therefore, it can be obtained:

(23) $$E_{f}\propto \frac{1}{\frac{\nu_{f}}{\nu_{0}}}=\frac{\nu_{0}}{\nu_{f}}$$

From Figure 14, it is easy to obtain:

(24) $$E_{f}\propto \frac{\nu_{0}}{\nu_{f}}=\frac{\frac{\nu_{0}}{\nu_{g}}}{\frac{\nu_{f}}{\nu_{g}}}=\frac{A+\alpha_{0}T}{\alpha_{f}\left(T-T_{\infty }\right)}=\frac{K_{1}}{T-T_{\infty }}+\frac{K_{2}}{1-\frac{T_{\infty }}{T}}$$

in which *A* is the value of *ν*_0_/*ν*_g_ at 0 K and *K*_1_ and *K*_2_ are constants.

As for *E_s_*, it must be related to the difficulty of internal rotation since different conformations of the polymer can be converted to each other through internal rotation of a single bond. Therefore, the interaction between monomers induced by the chemical structure certainly influences the motion of chain segments. Consequently, it can be obtained:

(25) $$E_{s}\propto \varepsilon$$

where *ε* stands for the structural barrier of internal rotation. Therefore, we have:

(26) $$E_{a}\approx \frac{K_{1}}{T-T_{\infty }}+\frac{K_{2}}{1-\frac{T_{\infty }}{T}}+\varepsilon$$

The first two items are determined by the free volume, and the last item is determined by the chemical structure. Near the glass transition, *T* is approximate to *T*_∞_. In this case, (*K*_1_/(*T* − *T*_∞_) + *K*_2_/(1 − *T*_∞_/*T*)) ≫ *ε*, indicating that *E_a_* strongly depends on *ν*_0_/*ν_f_*. That is the reason why Equation (20) can be used only in the glass transition region [46]. At high temperatures far the above glass transition, *T* ≫ *T*_∞_, meaning *K*_1_/(*T* − *T*_∞_) is very small and *K*_2_/(1 − *T*_∞_/*T*) is approximate to *K*_2_. In this case, *E_a_* strongly depends on *K*_2_ + *ε*, indicating that *E_a_* shows weak temperature dependence at high temperatures. This fact is consistent with the Arrhenius form (Equation (5)) in which the apparent activation energy is independent of temperature. Figure S9 (seen in the Supporting Information) shows the activation energies of PS and PMMA. It can be seen that the activation energy at low temperatures near the glass transition shows a strong temperature dependence, and at high temperatures, it shows a weak temperature dependence. This fact is consistent with the discussions above. On the other hand, as for the VFTH form (Equation (2)), it can be expressed as:

(27) $$\tau =\tau_{0}exp\frac{\frac{B}{1-\frac{T_{\infty }}{T}}}{T}$$

In view of the form of Equation (27) and item *K*_1_/(*T* − *T*_∞_) + *K*_2_/(1 − *T*_∞_/*T*), it is easy to understand that the apparent activation energy of the VFTH form only depends on *K*_2_/(1 − *T*_∞_/*T*). Obviously, that means that the value of *ν*_0_/*ν_g_* at 0 K is too small, leading to the rationality of ignoring the item *K*_1_/(*T* − *T*_∞_). In addition, it can be seen that the value of *T*_∞_ can regulate the temperature dependence of *E*_a_ according to Equation (26). When the value of *T*_∞_ is large, *E_a_* shows a strong temperature dependence; while *T*_∞_ is small, *E_a_* shows a weak temperature dependence. In the case of *T*_∞_ = 0, *E_a_* ≈ *K*_2_ + *ε*, showing the independence of temperature. In that case, Equation (27) possesses constant activation energy, equal to the Arrhenius form. Therefore, it can be revealed that the operation of *T*_∞_ = 0 in Section 3.1 is to regulate the temperature dependence of *E_a_*; in fact, resulting in the equivalence of the VFTH form to the Arrhenius form.

In addition, if the DWLF equation can be applicable in a wide temperature range from *T*_g_ to very high temperatures, its activation energy should show a similar temperature dependence of (*K*_2_/(1 − *T*_∞_/*T*) + *ε*) according to Equation (26) (item *K*_1_/(*T* − *T*_∞_) is ignored). Since Equation (11) can be expressed as:

(28) $$\Delta E_{a}^{\prime }=\frac{2.303RC_{1}^{\prime }C_{2}^{\prime }}{{\left(1-\frac{T_{s}-C_{2}^{\prime }}{T}\right)}^{2}}$$

and *R*, *C*_1_′, *C*_2_′ and *T_s_* are all constants, the temperature dependence of ∆${E_{a}}^{\prime }$ just depends on the item *X*_1_/(1 − *X*_2_/*T*)^2^. *X*_1_ and *X*_2_ are adjustable parameters. This form is similar to *K*_2_/(1 − *T*_∞_/*T*), meaning that *∆*$E_{a}^{\prime }$ can be approximate to *K*_2_/(1 − *T*_∞_/*T*) + *ε* and show a similar temperature dependence when appropriate values for *R*, *C*_1_′, *C*_2_′ and *T_s_* are adopted. As a result, the DWLF equation can be applied in wide temperature range.

In addition, if Equation (4) is introduced into Inequality (14), we have:

(29) $$0<T_{\infty }<T$$

That means that although the DWLF equation can be used over a wide temperature range, it should be used only above the equilibrium glass transition. This result is consistent with Equation (26). When *T* is below *T*_∞_, the first two items in Equation (26) representing the contribution of free volume are both negative values, which are meaningless. This fact indicates in the case of the application in a continuous wide temperature range, the DWLF equation can and must be applicable above the equilibrium glass transition to high temperatures. As for describing the secondary relaxations, the DWLF equation should be used only in the case of *T*_∞_ = 0, i.e., *C*_2_′ = *T_s_*. In that case, *E_a_* ≈ *K*_2_ + *ε* (according to Equation (26)), independent of temperature, and thus, the DWLF (or VFTH) equation is equivalent to the Arrhenius form. However, it should be pointed out that when *T*_∞_ = 0, although the DWLF equation is equivalent to the Arrhenius form and thus can describe the secondary relaxations just as shown in Figure 12 and Figure 13, this equation cannot be applicable at a wide temperature range since *E_a_* ≈ *K*_2_ + *ε* shows no temperature dependence.

Based on the above results and discussions, we propose a developed WLF equation that can be universal for polymers from glass transition to terminal relaxation and also applicable for secondary relaxations. Furthermore, we explain why the DWLF equation can achieve the time-temperature superposition of the relaxation properties of polymers in a wide temperature range theoretically. The Arrhenius form is the limit form of the DWLF equation or the VFTH equation. Based on this viewpoint, it is reasonable to draw the conclusion that there should be a universal essential mechanism for different relaxations of polymers.

## 4. Conclusions

The mathematical relationship between the three equations, i.e., the WLF equation, the VFTH equation and the Arrhenius equation, was discussed, and a developed WLF equation was proposed. Based on the different temperature-dependences of apparent activation energy between the WLF and Arrhenius forms, the necessary criterion for the universality of the DWLF form was proposed. Results show that at high temperatures far away from the glass transition, the DWLF equation can be valid to describe the temperature dependence of crystalline and amorphous polymers, including *i*PP, HDPE, LDPE and EPR provided *C*_2_′ = *T_s_*. The phase-separation behavior of PMMA/SMA and *i*PP/EPR blends can be described by the DWLF equation well, while the nucleation process leads to a smaller shift factor for the crystalline/amorphous blend in the melting temperature region. Through investigating TTS of PS and PMMA and secondary relaxations of PMMA and PVC, it was proven that the Arrhenius equation can be valid only in the high temperature region and invalid in the vicinity of glass transition due to the strong dependence of apparent activation energy on temperature; while the DWLF equation can be employed to the whole temperature region including secondary relaxation and from glass transition to terminal relaxation. Through discussing the influences of free volume and chemical structure on the activation energy of polymer relaxation in more detail, the reason for the universal application of the DWLF equation was revealed theoretically.

## Figures and Tables

**Table 1:** Fitting parameters at different reference temperatures for *i*PP. GOF, goodness of fit.

*C*_1_′	*C*_2_′ (K)	*T_s_* (K)	GOF
5.72	424	424	0.991
5.81	427	427	0.993
5.76	430	430	0.993
5.87	433	433	0.985
5.52	436	436	0.988
5.59	439	439	0.967
5.51	442	442	0.992
5.31	445	445	0.981
5.46	448	448	0.993
5.31	451	451	0.992
5.24	454	454	0.985
5.18	457	457	0.988
5.43	460	460	0.993
5.03	464	464	0.991
4.92	467	467	0.988
5.38	470	470	0.992
5.04	473	473	0.992
5.32	477	477	0.992
5.63	481	481	0.986
5.53	485	485	0.983
5.23	489	489	0.989
5.01	493	493	0.981
4.81	497	497	0.992
4.81	501	501	0.993
4.80	505	505	0.993
5.59	509	509	0.978
5.31	513	513	0.973
5.19	517	517	0.988
5.37	521	521	0.982
5.60	525	525	0.972

## Supplementary Materials

The supplementary materials are available online at www.mdpi.com/2073-4360/9/11/567/s1.
