# Supplementary material for: New Insight into Time-Temperature Correlation for Polymer Relaxations Ranging from Secondary Relaxation to Terminal Flow: Application of a Universal and Developed WLF Equation
Source: Polymers (Basel). 2017 Nov 2;9(11):567. doi: 10.3390/polym9110567 (PMC6418538; doi:10.3390/polym9110567)
Supplement: Supplementary file 1 [file polymers-09-00567-s001.doc]

**Supporting Information**

**New Insight into Time-Temperature Correlation for Polymer Relaxations Ranging from Secondary Relaxation to Terminal Flow: Application of a Universal and Developed
WLF Equation**

Yonggang Shangguan1[[1]](#footnote-2)*, Feng Chen^1^, Erwen Jia^1^, Yu Lin^1^, Jun Hu^2^, Qiang Zheng1[[2]](#footnote-3)*

*^1^MOE Key Laboratory of Macromolecular Synthesis and Functionalization, Department of Polymer Science and Engineering, Zhejiang University, Hangzhou 310027, P. R. China*

*^2^The Affiliated Stomatology Hospital, College of Medicine, Zhejiang University, Hangzhou, 310006, P. R. China.*

|  |  |
| --- | --- |
|  |  |
|  |  |
|  |  |
|  |  |
|  |  |
|  |  |
|  |  |
|  |  |
|  |  |
|  |  |
|  |  |
|  |  |
|  |  |
|  |  |
| **Figure S1.** Figures for the fitting results by the DWLF equation at different reference temperatures for *i*PP. | |

|  |
| --- |
| **Figure S2.** Glass transition temperature investigated by dynamic mechanical analysis (DMA) measurement for *i*PP. |

|  |  |
| --- | --- |
| **Figure S3.** Glass transition temperature investigated by DMA measurement for (**a**) HDPE and  (**b**) LDPE. | |

|  |
| --- |
| **Figure S4.** Glass transition temperature investigated by DMA measurement for EPR. |

|  |
| --- |
| **Figure S5.** Melting curve of *i*PP investigated by DSC. |

|  |
| --- |
| **Figure S6.** Glass transition temperature investigated by DSC measurement for PS. |

|  |
| --- |
| **Figure S7.** Glass transition temperature investigated by DSC measurement for PMMA. |

|  |  |
| --- | --- |
| **Figure S8.** (**a**) DMA result of PVC and (**b**) the β-relaxation process of PVC measured by DMA at different frequencies. | |

|  |
| --- |
| **Figure S9.** Activation energies of PS and PMMA. |

**Mathematical Derivation of Equation (3) and Equation (6)**

The WLF equation and the VFTH equation are in the following form:

$\text{lg}\alpha_{T}=\text{-}\frac{C_{1}\left(T-T_{s}\right)}{C_{2}+\left(T-T_{s}\right)}$ (1)

$\tau=\tau_{0}\exp\frac{B}{T-T_{\infty}}$ (2)

Since *α*_T_ is the ratio of two relaxation times, which can be expressed as Equation (2), the relaxation times at arbitrary *T* and reference temperature *T*_s_ can be written as:

$$\tau=\tau_{0}\exp\frac{B}{T-T_{\infty}}$$

$$\tau_{s}=\tau_{0}\exp\frac{B}{T_{s}-T_{\infty}}$$

The shift factor *α*_T is_ equal to the ratio of *τ* and *τ_s_*:

$$\alpha_{T}=\text{-}\frac{\exp\frac{B}{T-T_{\infty}}}{\exp\frac{B}{T_{s}-T_{\infty}}}=\exp[\frac{B}{T-T_{\infty}}-\frac{B}{T_{s}-T_{\infty}}]$$

Thus, we can obtain the lg*α*_T_ form of the VFTH equation:

$$\begin{matrix} \text{lg}\alpha_{T}=\frac{1}{2.303}[\frac{B}{T-T_{\infty}}-\frac{B}{T_{s}-T_{\infty}}]=\frac{B}{2.303}[\frac{1}{T-T_{\infty}}-\frac{1}{T_{s}-T_{\infty}}] \\ =\frac{B}{2.303}[\frac{(T_{s}-T_{\infty})-(T-T_{\infty})}{(T-T_{\infty})(T_{s}-T_{\infty})}]=\frac{B}{2.303}[\frac{(T_{s}-T)}{(T-T_{\infty})(T_{s}-T_{\infty})}] \\ =\frac{B}{2.303}(T_{s}-T_{\infty})[\frac{(T_{s}-T)}{(T-T_{\infty})}]=\frac{B}{2.303}(T_{s}-T_{\infty})[\frac{(T_{s}-T)}{(T_{s}-T_{\infty}+T-T_{s})}] \end{matrix}$$

Compared with the WLF form of Equation (1), we can obtain:

$C_{1}=\frac{B}{2.303(T_{s}-T_{\infty})}$ (3)

$C_{2}=T_{s}-T_{\infty}$ (4)

The Arrhenius form is written as:

$\tau=\tau_{0}\exp\frac{E_{a}}{RT}$ (5)

Similar to the lg*α*_T_ form of the VFTH equation, we can obtain the relaxation times at arbitrary *T* and reference temperature *T*_s_:

$$\tau=\tau_{0}\exp\frac{E_{a}}{RT}$$

$$\tau_{s}=\tau_{0}\exp\frac{E_{a}}{RT_{s}}$$

Therefore, the lg*α*_T_ form of the Arrhenius equation can be written as:

$$\begin{matrix} \text{lg}\alpha_{T}=\frac{1}{2.303}[\frac{E_{a}}{RT}-\frac{E_{a}}{RT_{s}}]=\frac{E_{a}}{2.303R}[\frac{1}{T}-\frac{1}{T_{s}}]=\frac{0.434E_{a}}{R}[\frac{T_{s}-T}{TT_{s}}] \\ =0.434\frac{E_{a}}{RT_{s}}[\frac{T_{s}-T}{T}]=-0.434\frac{E_{a}}{RT_{s}}[\frac{T-T_{s}}{T}]=\frac{-0.434\frac{E_{a}}{RT_{s}}[T-T_{s}]}{T} \end{matrix}$$

$\text{lg}\alpha_{T}=\text{-}\frac{0.434\frac{E_{a}}{RT_{s}}\left(T-T_{s}\right)}{T_{s}+\left(T-T_{s}\right)}$ (6)

1. ^*^ Corresponding author, E-mail: [shangguan@zju.edu.cn](mailto:shangguan@zju.edu.cn); [zhengqiang@zju.edu.cn](mailto:zhengqiang@zju.edu.cn) [↑](#footnote-ref-2)
2. ^*^ [↑](#footnote-ref-3)
